# Supplementary material for: Early intervention services for non-psychotic mental health disorders: a scoping review protocol
Source: BMJ Open. 2019 Dec 5;9(12):e033656. doi: 10.1136/bmjopen-2019-033656 (PMC6924732; doi:10.1136/bmjopen-2019-033656)
Supplement: Author's manuscript [file bmjopen-2019-033656.draft_revisions.pdf]

BMJ Open is committed to open peer review. As part of this commitment we make the peer review history of every article we publish publicly available.

When an article is published we post the peer reviewers' comments and the authors' responses online. We also post the versions of the paper that were used during peer review. These are the versions that the peer review comments apply to.

The versions of the paper that follow are the versions that were submitted during the peer review process. They are not the versions of record or the final published versions. They should not be cited or distributed as the published version of this manuscript.

BMJ Open is an open access journal and the full, final, typeset and author-corrected version of record of the manuscript is available on our site with no access controls, subscription charges or pay-per-view fees (http://bmjopen.bmj.com).

If you have any questions on BMJ Open's open peer review process please email info.bmjopen@bmj.com

BMJ Open

Early intervention services in mental health: a scoping review protocol

Journal:	*BMJ Open*
Manuscript ID	bmjopen-2019-033656
Article Type:	Protocol
Date Submitted by the Author:	15-Aug-2019
Complete List of Authors:	Richards, Katie; King's College London, Psychological Medicine<br>Austin, Amelia; King's College London, Psychological Medicine<br>Allen, Karina; South London and Maudsley Mental Health NHS Trust; King's College London, Psychological Medicine<br>Schmidt, Ulrike; King's College London, Psychological Medicine; South London and Maudsley Mental Health NHS Trust
Keywords:	Early Intervention, Implementation, MENTAL HEALTH, Health Service Design

SCHOLARONE™
Manuscripts

Early intervention services in mental health: a scoping review protocol

Authors: Katie Richards[1], Amelia Austin[1], Karina Allen[1,2,3], & Ulrike Schmidt[1,2]

1: King's College London, Institute of Psychiatry, Psychology and Neuroscience, London, UK

2: South London and Maudsley NHS Foundation Trust, London, UK

3: School of Psychological Science, The University of Western Australia

ORCID: KR 0000-0003-3826-6317, AA 0000-0002-4979-4847, KA 0000-0003-2896-6459, US 0000-0003-1335-1937

Corresponding author:

Katie Richards, Eating Disorders Unit, Institute of Psychiatry, Psychology, and Neuroscience, King's College London, Box PO59, De Crespigny Park, London SE5 8AF, katie.richards@kcl.ac.uk

Word count: 2,072 (excludes the title page, abstract, tables, acknowledgements, contributions and references)

For peer review only

ABSTRACT

Introduction: Worldwide mental health disorders are associated with a considerable amount of human suffering, disability and mortality. Yet, the provision of rapid evidence-based care to mitigate the human and economic costs of these disorders is limited. The greatest progress in developing and delivering early intervention services has occurred within psychosis. There is now growing support for and calls to extend such approaches to other diagnostic groups. The aim of this scoping review is to systematically map this emerging literature on early intervention services for non-psychotic mental health disorders, with a focus on outlining how services are structured, implemented, and scaled. **Methods and analysis:** The protocol was developed using the guidance for scoping reviews in the Joanna Briggs Institute manual. A systematic search for published and unpublished literature will be conducted using the following databases: (1) Medline; (2) PsycINFO; (3) HMIC; (4) Embase; and (5) ProQuest. To be included, documents must describe and/or evaluate an early intervention service for adolescents or adults with a non-psychotic mental health disorder. There will be no restrictions on publication type, study design, and date. Title and abstract, and full-text screening will be completed by one reviewer, with a proportion of articles screened in duplicate. Data analysis will primarily involve a qualitatively summary of the included articles exploring the characteristics of early intervention services and barriers and facilitators to implementation. **Ethics and dissemination:** The synthesis of published and unpublished articles will not require ethical approval. The results of this scoping review will be published in a peer-reviewed journal and disseminated via social media, conference presentations, and other knowledge translation activities.

Keywords: early intervention, mental health disorders, service design, implementation

For peer review only

ARTICLE SUMMARY

Strengths and limitations of this study

- This scoping review will provide a comprehensive overview of both published and unpublished literature for the emerging research field of early intervention services for non-psychotic mental health disorders.
- The review will be conducted according to the standardised methodology outlined in the Joanna Briggs Institute manual.
- Part of the screening and charting process will be completed in duplicate to ensure reliability of these methods.
- Only articles written in English, German, French, and Spanish will be included, the review may therefore be biased.

For peer review only

INTRODUCTION

Early intervention is widely perceived as beneficial in medicine and refers to the early detection and initiation of stage-specific treatment.[1] Pro-active treatments matched to the stage of illness can limit or even avert unfavourable outcomes, reducing the need for costly and more invasive treatments in the future.[2,3] Despite such promise, early intervention approaches have been slow to gain momentum in mental health.[4,5] Mental illnesses are a major contributor to mortality and disability worldwide, particularly for young people.[6-8] The typical age of onset for mental disorders is adolescence and early adulthood (12-30 years old), a period of marked social, psychological, and biological change.[9,10] A delay in or lack of access to effective treatments during this time could disrupt key developmental milestones and have long-lasting effects on health, social, and occupational trajectories.[11]

Service provision does not match the topography of onset or burden of disease associated with mental disorders, even in relatively well-developed mental health systems.[12] Globally, access to evidence-based care is poor, and even for those that do access it, this is often after lengthy delays.[13-15] The duration of untreated illness (DUI), defined as the period between the onset of psychiatric disorder and the initiation of treatment, ranges from 1-2 years for psychosis to 10 years for obsessive-compulsive disorder (OCD).[16-19] Over time, mental disorders may become more entrenched through functional deterioration, neuroadaptation, and habitual behaviour patterns.[20-23] Indeed, a longer DUI is associated with worse symptomatic and functional outcomes and a lower treatment response across diagnostic groups.[19,24-27] More worryingly, young people, the group at highest risk for psychiatric difficulties, tend to have the worst access to timely care.[13,18,28-30]

Together, such findings provide a strong case for establishing early intervention services that match the developmental needs and symptomatic profile of individuals with recent-onset mental disorders.[4,14] Over the past 30 years, early intervention for psychosis (EIP) has gained tremendous support from researchers and healthcare professionals worldwide.[14] A comprehensive body of high-quality research now shows that compared to standard care, integrated multi-component EIP services are associated with a reduction in symptom severity, relapse rates and hospitalisation risk, as well as improved global functioning and quality of life.[31] Moreover, consistent evidence suggests that across different settings EIP services are a cost-effective alternative to standard care.[32] In contrast, there is a paucity of research evaluating early intervention services for other mental health diagnoses. There has been a recent surge in papers calling for early intervention approaches

For peer review only

to be broadened to other areas of mental health, including major depression,[33] OCD,[22] eating disorders,[34] and bipolar disorder.[35]

The role of early intervention in reducing distress and functional impairment in mental health seems obvious.[14,22] However, much more work needs to be done. There is limited prospective evidence evaluating the utility and impact of early intervention services in non-psychotic disorders. It is unclear to what extent the findings from psychosis would translate to other diagnostic groups. Moreover, even within psychosis, further research is needed to determine how long EIP services should be provided, whether it is the reduction in DUI or other components of EIP services that account for the improved outcomes, and whether outcomes would be similar with other service structures and models.[36,37] An ever-growing population accompanied by reducing health budgets, creates a ruthless environment where only services that demonstrate effectiveness, economic viability and sustainability receive funding.[38] It is therefore imperative to develop a rigorous evidence-base to refine, adapt and evaluate early intervention services for non-psychotic disorders, with a particular focus on identifying the "active ingredients" of such services and the most effective methods for widespread scaling and implementation.

The primary objective of this review is to identify and describe the differing ways in which early intervention services are structured and implemented for non-psychotic mental health disorders. The emerging literature for non-psychotic disorders is heterogenous and dispersed, with distinct streams of research developing in disciplinary silos. The aim of this review is to draw together these streams to facilitate collaboration and cross-disciplinary learning and discourse. By synthesising the field and highlighting commonalities and differences, we hope that a broad set of common principles for early intervention services will emerge. This review, in conjunction with reviews in psychosis, will help set the stage for a more unified approach to expanding and refining early intervention services for psychiatric disorders. Here, we focus exclusively on disorders that tend to emerge in adolescence and adulthood rather than in childhood, as neurodevelopmental disorders typically use a very different approach to early intervention (e.g. intervening in infancy).[39] A scoping review methodology was selected for this review as early intervention research is dispersed and heterogenous and therefore is not amenable to the narrower aims of a traditional systematic review.[40,41] Given that this is a relatively new research area, we sought to map all the available evidence within this field rather than only the best available evidence (e.g. randomised controlled trials).[42]

For peer review only

RESEARCH QUESTIONS

- What is the extent, range, and nature of the literature on early intervention services for adolescents and adults with non-psychotic mental health disorders?
- What are the characteristics of these early intervention services and care pathways?
    - Are there any similarities and differences across diagnoses and the types of early intervention services?
    - Are there any facilitators and/or barriers to implementing early intervention services?
- Do early intervention services reduce DUI, improve the course and outcome of mental disorders or minimise the disruption to psychosocial development and function?

METHODS AND ANALYSIS

The PRISMA extension for scoping reviews (PRISMA-ScR),[41] and the scoping review framework outlined in the Joanna Briggs Institute Reviewer's Manual[43] were used to guide the development of this protocol.

Eligibility criteria

We will include documents that describe and/or evaluate early intervention services for non-psychotic mental health disorders (*concept*). Early intervention services based in any type of healthcare facility, including hospitals, day services, and community settings, and any geographic area will be eligible for inclusion (*context*). Documents will be included if they describe and/or evaluate an early intervention service for adolescents (≥ 10-17 years) or adults (> 18 years) with a recent-onset mood disorder, anxiety disorder, eating disorder, personality disorder, impulse control or substance use disorder, and/or somatoform disorder (*types of participants*). Early intervention services for comorbid disorders will be included provided that at least one of the diagnoses listed in the previous sentence is of equal or greater interest than the non-listed condition. Mixed child and adolescent services will also be included, but only information relevant for the adolescent portion will be charted. All types of studies will be included: randomised controlled trials, non-randomised studies, qualitative studies, ongoing trials, protocols, editorials, opinions, and expert consensus statements (*types of studies*).

Documents will be excluded if they describe a primary prevention programme based in a school or in the general population, a parental intervention, describe a specific intervention (e.g. type of CBT) that is not attached to a service, or the diagnosis of interest is a physiological or medical condition (e.g. depression in the context of cancer), schizophrenia spectrum and other psychotic disorders, and/or neurodevelopmental disorders.

For peer review only

Search strategy

A comprehensive literature search will be conducted from inception on PsycINFO, MEDLINE, Embase, and HMIC. ProQuest databases will also be searched for grey literature (i.e. conference papers and proceedings, theses, government publications). The search is completed in three stages. First, an initial limited search was conducted in MEDLINE using the terms "early intervention" and "mood disorder" or "anxiety disorder" or "eating disorder" or "personality disorder" or "impulse control disorder" or "substance use disorder" or "somatoform disorder". The keywords and subject headings identified in this initial search were used to develop a search strategy. The MEDLINE-specific search strategy returns 12,363 documents before de-duplication (10,533 following de-duplication) and is outlined in Table 1. An iterative process was used to develop this search strategy balancing sensitivity and specificity, and ensuring key articles were returned.

In the second stage, all databases will be searched using the MEDLINE search strategy tailored to each database. The search for scoping reviews is iterative, as the reviewers become familiar with the literature, it is therefore possible that additional search terms and sources may be identified. The final stage involves identifying additional articles by searching the reference lists of included articles. Studies not reported in English, German, French, and Spanish will be excluded from the review during the screening and eligibility assessment. No date limits will be applied to the search. References will be imported to the EndNote x8 reference manager.

Table 1. MEDLINE search strategy

	**Query**	**Results**
#1	exp Early Medical Intervention [MeSH term]/ or ((early adj1 (intervent* or treat* or recogni* or detect* or service*)) or ((first or initial) adj1 (admission* or hospital* or episode*)) or (early-intervention* AND service*) or (early intervention* AND service*)).tw.	142144
#2	exp Mood Disorders [MeSH term]/ or Bipolar Disorders [MeSH term]/ or (mood disorder* or affective disorder* or depressi* or dysthymi* or bipolar*).tw	448227
#3	#1 AND #2	6521
#4	exp Anxiety Disorders [MeSH term]/ or (anxiety disorder* or neurotic disorder* or agoraphobi* or obsessive-compulsive disorder* or OCD or panic disorder* or phobic disorder* or post-traumatic stress	118524

For peer review only

	disorder* or post traumatic stress disorder* or PTSD or generalised anxiety disorder* or social phobia).tw	
#5	#1 AND #4	1390
#6	exp "Feeding and Eating Disorders" [MeSH term]/ or (eating disorder* or anorexi* or bulimi* or binge-eating* or binge eating* or (eating disorder not otherwise specified) or EDNOS or (other specified feeding or eating disorder) or OSFED).tw	56037
#7	#1 AND #6	662
#8	exp Substance-Related Disorders [MeSH term]/ or exp "Disruptive, Impulse Control, and Conduct Disorders" [MeSH term]/ or (((substance-related or alcohol or opioid or morphine or marijuana or heroin or cocaine or amphetamine or cannabis) adj1 (disorder* or illness* or dependence or abuse or misuse)) or (impulse control disorder*) or conduct disorder* or fire setting behaviour* or gambling or trichotillomania).tw	293156
#9	#1 AND #8	2864
#10	exp Somatoform Disorders [MeSH term]/ or (somatoform or somatoform disorder* or somati#ation or body dysmorphi* or conversion disorder* or hypochondri*).tw	2595
#11	#1 AND #10	225
#12	exp Personality Disorders [MeSH terms]/ or (personality disorder* or antisocial personality disorder* or anti-social personality disorder* or borderline personality disorder* or emotionally unstable personality disorder* or obsessive-compulsive personality disorder* or dependent personality disorder* or histrionic personality disorder* or narcissistic personality disorder* or avoidant personality disorder* or paranoid personality disorder* or schizoid personality disorder* OR schizotypal personality disorder*).tw	46823
#13	#1 AND #12	701

Study selection process

The initial limited search was conducted by KR in April 2019 to generate a preliminary search strategy. The preliminary search strategy was reviewed by AA, KA, and US, and modifications were made to improve the specificity of the search. The title and abstract screening in the

For peer review only

second stage will be completed by one reviewer, and a portion of the articles will be screened in duplicate to ensure reliability (25%). Retrieved full-texts will be screened by one reviewer and a sample of full-text documents (25%) will be screened in duplicate. Discrepancies will be resolved by discussion and if necessary other members of the review team will be consulted.

Data items and charting

A standardised data charting form developed by the study team will be used to chart the data from eligible studies (Table 2). The following data items are included on the form: document characteristics (e.g. author(s), publication date), study design (if applicable), the aim of the paper, population/participants, context, service characteristics, comparator/standard care characteristics (if applicable), outcomes (if applicable), primary findings (if applicable), and facilitators and barriers to early intervention services (if applicable). Similar to the full-text screening, one reviewer will chart the majority of the documents with only a portion (25%) of the documents being charted in duplicate to ensure reliability. Where there is more than one paper on the same service model, information will be pooled across the papers to provide the most detailed description of the model and any available evidence.

Table 2. Draft data charting form

**Study Details and Characteristics**	
Author(s)	
Year of Publication	
Title	
Journal	
Country of Origin	
Type of Study/Article (e.g. intervention trial, opinion)	
Study Design (if applicable)	
Aim/Purpose	
Population (e.g. target age, diagnosis) & Participants (if applicable) (e.g. number, sex, age)	
Context (healthcare setting (e.g. inpatient vs outpatient); geographical setting (e.g. rural vs urban))	

For peer review only

Details of Early Intervention Service (specifically: prioritisation, access, approach, care model (e.g. stand-alone specialist multidisciplinary service, specialist multidisciplinary team within a general mental health team, satellite team embedded in another service, enhanced community mental health teams), engagement, assessment, treatment)	
Details of Comparator or Standard Care (if applicable)	
**Results Extracted from Study**	
Outcomes (if applicable)	
Primary Findings/Conclusions	
Facilitators to Early Intervention Service (either observed or anticipated)	
Barriers to Early Intervention Service (either observed or anticipated)	

Critical appraisal

The lack of critical appraisal tools in scoping reviews has been highlighted as one of the primary limitations of this knowledge synthesis method.[44] Critical appraisal can facilitate the interpretation of reviews by identifying the relative strengths and weaknesses of the included articles and identifying gaps in the research field. However, formal evaluations of methodological quality for scoping reviews can be challenging given the diversity of study designs and the volume of included literature.[45] Given the range of study designs, a two-stage assessment of methodological quality will be conducted for this review. First, each study will be ranked using the Joanna Briggs Institutes Levels of Evidence for Effectiveness from high (Level 1) to low (Level 5) (Level 1 – Experimental Designs; Level 2 – Quasi-experimental Designs; Level 3 – Observational - Analytical; Level 4 – Observational - Descriptive; Level 5 – Expert Opinion and Bench Research).[46] Once stratified according to the level of evidence, the quality of the studies within each stratum will be evaluated using the Joanna Briggs Institute Critical Appraisal tools.[47] A narrative summary of the methodological quality will be provided. A portion of the included articles will be appraised in duplicate.

Synthesis of results

The search results will be reported using a flow diagram to clearly detail the review decision process, indicating the number of citations screened, duplicates removed, study selection, and full texts retrieved. The characteristics of the included studies will be presented in an informative table with a narrative and quantitative (e.g. frequencies) summary in text. Figures

For peer review only

will be used to display the distribution of documents over time and across diagnoses. Descriptions of the early intervention services will be reported for each diagnostic group along with any evidence supporting the services and barriers and facilitators to implementation. An aggregated summary of early intervention services with descriptions of common themes and differences across the services will be provided. An effort will be made to identify gaps in knowledge to inform the direction of future research.

Patient and public involvement

No patients or public were involved in the development of this protocol.

ETHICS AND DISSEMINATION

This review contributes to the growing body of research for early intervention initiatives in mental health by mapping the existing literature on early intervention services for non-psychotic mental health disorders. Through the publication of the results and dissemination via social media and conference presentations, the results will hopefully provide a timely foundation for cross-disciplinary discourse and early intervention service development and research. The results of this review may inform the design of new services and policies to support them. The synthesis of existing knowledge will not require ethical approval.

Author contributions All authors contributed to the development of this protocol. KR drafted the manuscript and search strategy. AA, KA, and US reviewed the search strategy and the draft manuscripts. KR incorporated the feedback from the authors. All authors read and approved the final manuscript.

Funding statement KR and AA are supported by the Health Foundation Scaling Up programme. US is supported by a National Institute of Health Research (NIHR) Senior Investigator Award and receives salary support from the NIHR Mental Health Biomedical Research Centre at the South London and Maudsley NHS Foundation Trust and King's College London. KA is supported by the NHS Innovation Accelerator programme. The views expressed are those of the author(s) and not necessarily those of the NHS, the NIHR or the Department of Health.

Competing interest statement None declared.

Patient consent Not required.

For peer review only

REFERENCES

1. McGorry PD. Is early intervention in the major psychiatric disorders justified? Yes. *BMJ* 2008;337:a695. doi: 10.1136/bmj.a695
2. Gillies CL, Abrams KR, Lambert PC, et al. Pharmacological and lifestyle interventions to prevent or delay type 2 diabetes in people with impaired glucose tolerance: systematic review and meta-analysis. *BMJ* 2007;334(7588):299. doi: 10.1136/bmj.39063.689375.55
3. Shah C, Arthur DW, Wazer D, et al. The impact of early detection and intervention of breast cancer-related lymphedema: a systematic review. *Cancer Med J* 2016;5(6):1154-62. doi: 10.1002/cam4.691
4. McGorry PD, Mei C. Early intervention in youth mental health: progress and future directions. *Evid Based Ment Health* 2018;21(4):182. doi: 10.1136/ebmental-2018-300060
5. McGorry PD, Ratheesh A, O'Donoghue B. Early Intervention—An Implementation Challenge for 21st Century Mental Health CareEarly Intervention—An Implementation Challenge for 21st Century Mental Health CareEditorial. *JAMA Psychiatry* 2018;75(6):545-46. doi: 10.1001/jamapsychiatry.2018.0621
6. Patel V, Flisher AJ, Hetrick S, et al. Mental health of young people: a global public-health challenge. *Lancet (London, England)* 2007;369(9569):1302-13. doi: 10.1016/S0140-6736(07)60368-7
7. Rehm J, Shield KD. Global Burden of Disease and the Impact of Mental and Addictive Disorders. *Curr Psychiatry Rep* 2019;21(2):10. doi: 10.1007/s11920-019-0997-0
8. Vigo D, Thornicroft G, Atun R. Estimating the true global burden of mental illness. *Lancet Psychiatry* 2016;3(2):171-78. doi: 10.1016/S2215-0366(15)00505-2
9. de Girolamo G, McGorry PD, Sartorius N, editors. *Age of Onset of Mental Disorders*. Switzerland: Springer International, 2019.
10. Kessler RC, Angermeyer M, Anthony JC, et al. Lifetime prevalence and age-of-onset distributions of mental disorders in the World Health Organization's World Mental Health Survey Initiative. *World J Psychiatry* 2007;6(3):168-76.
11. Malla A, Shah J, Iyer S, et al. Youth Mental Health Should Be a Top Priority for Health Care in Canada. *Can J Psychiatry* 2018;63(4):216-22. doi: 10.1177/0706743718758968
12. Vigo DV, Kestel D, Pendakur K, et al. Disease burden and government spending on mental, neurological, and substance use disorders, and self-harm: cross-sectional, ecological study of health system response in the Americas. *Lancet Public Health* 2019;4(2):e89-e96. doi: 10.1016/S2468-2667(18)30203-2
13. de Girolamo G, Dagani J, Purcell R, et al. Age of onset of mental disorders and use of mental health services: needs, opportunities and obstacles. *Epidemiol Psychiatric Sci* 2012;21(1):47-57. doi: 10.1017/S2045796011000746
14. McGorry PD. Early intervention in psychosis: obvious, effective, overdue. *J Nerv Ment Dis* 2015;203(5):310. doi: 10.1097/NMD.0000000000000284
15. Patel V, Saxena S, Lund C, et al. The Lancet Commission on global mental health and sustainable development. *Lancet (London, England)* 2018;392(10157):1553-98. doi: 10.1016/S0140-6736(18)31612-X
16. Albert U, Barbaro F, Bramante S, et al. Duration of untreated illness and response to SRI treatment in Obsessive-Compulsive Disorder. *Euro Psychiatry* 2019;58:19-26. doi: 10.1016/j.eurpsy.2019.01.017
17. Altamura AC, Buoli M, Albano A, et al. Age at onset and latency to treatment (duration of untreated illness) in patients with mood and anxiety disorders: a naturalistic study. *Int Clin Psychopharmacol* 2010;25(3):172-79.

For peer review only

18. Dagani J, Signorini G, Nielssen O, et al. Meta-analysis of the Interval between the Onset and Management of Bipolar Disorder. *Can J Psychiatry* 2017;62(4):247-58. doi: 10.1177/0706743716656607
19. Marshall M, Lewis S, Lockwood A, et al. Association Between Duration of Untreated Psychosis and Outcome in Cohorts of First-Episode Patients: A Systematic Review. *JAMA Psychiatry* 2005;62(9):975-83. doi: 10.1001/archpsyc.62.9.975
20. Anderson KK, Voineskos A, Mulsant BH, et al. The Role of Untreated Psychosis in Neurodegeneration: A Review of Hypothesized Mechanisms of Neurotoxicity in First-Episode Psychosis. *Can J Psychiatry* 2014;59(10):513-17. doi: 10.1177/070674371405901003
21. Currin L, Schmidt U. A critical analysis of the utility of an early intervention approach in the eating disorders. *J Ment Health* 2005;14(6):611-24. doi: 10.1080/09638230500347939
22. Fineberg NA, Dell'Osso B, Albert U, et al. Early intervention for obsessive compulsive disorder: An expert consensus statement. *Eur Neuropsychopharmacol* 2019;29(4):549-65. doi: 10.1016/j.euroneuro.2019.02.002
23. Schaffalitzky E, Leahy D, Armstrong C, et al. 'Nobody really gets it': a qualitative exploration of youth mental health in deprived urban areas. *Early Interv Psychiatry* 2015;9(5):406-11. doi: 10.1111/eip.12165
24. Altamura AC, Dell'Osso B, D'Urso N, et al. Duration of Untreated Illness as a Predictor of Treatment Response and Clinical Course in Generalized Anxiety Disorder. *CNS Spectr* 2008;13(5):415-22. doi: 10.1017/S1092852900016588 [published Online First: 11/07]
25. Altamura AC, Dell'Osso B, Berlin HA, et al. Duration of untreated illness and suicide in bipolar disorder: a naturalistic study. *Eur Arch Psychiatry Clin Neurosci* 2010;260(5):385-91.
26. Drancourt N, Etain B, Lajnef M, et al. Duration of untreated bipolar disorder: missed opportunities on the long road to optimal treatment. *Acta Psychiatr Scand* 2013;127(2):136-44. doi: 10.1111/j.1600-0447.2012.01917.x
27. Ghio L, Gotelli S, Cervetti A, et al. Duration of untreated depression influences clinical outcomes and disability. *J Affect Disord* 2015;175:224-28. doi: 10.1016/j.jad.2015.01.014
28. Cleary A, Nixon E, Fitzgerald M. Psychological health and well-being among young Irish adults. *Ir J Psychol Med* 2007;24(4):139-44. doi: 10.1017/S0790966700010569 [published Online First: 06/13]
29. Burgess PM, Pirkis JE, Slade TN, et al. Service use for mental health problems: findings from the 2007 National Survey of Mental Health and Wellbeing. *Aust N Z J Psychiatry* 2009;43(7):615-23. doi: 10.1080/00048670902970858
30. Weigel A, Rossi M, Wendt H, et al. Duration of untreated illness and predictors of late treatment initiation in anorexia nervosa. *J Public Health* 2014;22(6):519-27. doi: 10.1007/s10389-014-0642-7
31. Correll CU, Galling B, Pawar A, et al. Comparison of Early Intervention Services vs Treatment as Usual for Early-Phase Psychosis: A Systematic Review, Meta-analysis, and Meta-regression. *JAMA Psychiatry* 2018;75(6):555-65. doi: 10.1001/jamapsychiatry.2018.0623
32. Aceituno D, Vera N, Prina AM, et al. Cost-effectiveness of early intervention in psychosis: systematic review. *The British journal of psychiatry : the journal of mental science* 2019;215(1):388-94. doi: 10.1192/bjp.2018.298 [published Online First: 2019/01/31]

For peer review only

33. Davey CG, McGorry PD. Early intervention for depression in young people: a blind spot in mental health care. *Lancet Psychiatry* 2019;6(3):267-72. doi: 10.1016/S2215-0366(18)30292-X
34. Schmidt U, Brown A, McClelland J, et al. Will a comprehensive, person-centered, team-based early intervention approach to first episode illness improve outcomes in eating disorders? *The International journal of eating disorders* 2016;49(4):374-77. doi: 10.1002/eat.22519
35. Vieta E, Salagre E, Grande I, et al. Early Intervention in Bipolar Disorder. *Am J Psychiatry* 2018;175(5):411-26. doi: 10.1176/appi.ajp.2017.17090972
36. Behan C, Masterson S, Clarke M. Systematic review of the evidence for service models delivering early intervention in psychosis outside the stand-alone centre. *Early Interv Psychiatry* 2017;11(1):3-13. doi: 10.1111/eip.12334
37. Fusar-Poli P, McGorry PD, Kane JM. Improving outcomes of first-episode psychosis: an overview. *World J Psychiatry* 2017;16(3):251-65. doi: 10.1002/wps.20446
38. Stuckler D, Reeves A, Loopstra R, et al. Austerity and health: the impact in the UK and Europe. *Eur J Public Health* 2017;27(4):18-21. doi: 10.1093/eurpub/ckx167
39. Cioni G, Inguaggiato E, Sgandurra G. Early intervention in neurodevelopmental disorders: underlying neural mechanisms. *Dev Med Child Neurol* 2016;58(4):61-66. doi: 10.1111/dmcn.13050
40. Peters MDJ, Godfrey CM, Khalil H, et al. Guidance for conducting systematic scoping reviews. *Int J Evid Based Healthc* 2015;13(3):141-46. doi: 10.1097/XEB.0000000000000050
41. Tricco AC, Lillie E, Zarin W, et al. PRISMA Extension for Scoping Reviews (PRISMA-ScR): Checklist and ExplanationThe PRISMA-ScR Statement. *Ann Intern Med* 2018;169(7):467-73. doi: 10.7326/m18-0850
42. Murray A, Daines L, Archibald D, et al. The relationship and effects of golf on physical and mental health: a scoping review protocol. *Br J Sports Med* 2016;50(11):647. doi: 10.1136/bjsports-2015-095914
43. Peters MDJ, Godfrey C, McInerney P, et al. Chapter 11: Scoping Reviews. In: Aromataris E, Munn Z, eds. Joanna Briggs Institute Reviewer's Manual: The Joanna Briggs Institute, 2017.
44. Pham MT, Rajić A, Greig JD, et al. A scoping review of scoping reviews: advancing the approach and enhancing the consistency. *Res Synth Methods* 2014;5(4):371-85. doi: 10.1002/jrsm.1123
45. Levac D, Colquhoun H, O'Brien KK. Scoping studies: advancing the methodology. *Implement Sci* 2010;5(1):69. doi: 10.1186/1748-5908-5-69
46. Jordan Z, Lockwood C, Aromataris E, et al. The updated JBI model for evidence-based healthcare. 2016 [Available from: https://joannabriggs.org/jbi-approach.html#tabbed-nav=Levels-of-Evidence.
47. The Joanna Briggs Institute. Joanna Briggs Institute Critical Appraisal tools 2017 [Available from: https://joannabriggs.org/critical_appraisal_tools.

For peer review only

BMJ Open

Early intervention services for non-psychotic mental health disorders: a scoping review protocol

Journal:	*BMJ Open*
Manuscript ID	bmjopen-2019-033656.R1
Article Type:	Protocol
Date Submitted by the Author:	02-Oct-2019
Complete List of Authors:	Richards, Katie; King's College London, Psychological Medicine<br>Austin, Amelia; King's College London, Psychological Medicine<br>Allen, Karina; South London and Maudsley Mental Health NHS Trust; King's College London, Psychological Medicine<br>Schmidt, Ulrike; King's College London, Psychological Medicine; South London and Maudsley Mental Health NHS Trust
**Primary Subject Heading**:	Mental health
Secondary Subject Heading:	Health services research, Mental health
Keywords:	Early Intervention, Implementation, MENTAL HEALTH, Health Service Design

SCHOLARONE™
Manuscripts

Early intervention services for non-psychotic mental health disorders: a scoping review protocol

Authors: Katie Richards[1], Amelia Austin[1], Karina Allen[1,2,3], & Ulrike Schmidt[1,2]

1: King's College London, Institute of Psychiatry, Psychology and Neuroscience, London, UK

2: South London and Maudsley NHS Foundation Trust, London, UK

3: School of Psychological Science, The University of Western Australia

ORCID: KR 0000-0003-3826-6317, AA 0000-0002-4979-4847, KA 0000-0003-2896-6459, US 0000-0003-1335-1937

Corresponding author:

Katie Richards, Eating Disorders Unit, Institute of Psychiatry, Psychology, and Neuroscience, King's College London, Box PO59, De Crespigny Park, London SE5 8AF, katie.richards@kcl.ac.uk

Word count: 2,725 (excludes the title page, abstract, tables, acknowledgements, contributions and references)

For peer review only

ABSTRACT

Introduction: Worldwide mental health disorders are associated with a considerable amount of human suffering, disability and mortality. Yet, the provision of rapid evidence-based care to mitigate the human and economic costs of these disorders is limited. The greatest progress in developing and delivering early intervention services has occurred within psychosis. There is now growing support for and calls to extend such approaches to other diagnostic groups. The aim of this scoping review is to systematically map the emerging literature on early intervention services for non-psychotic mental health disorders, with a focus on outlining how services are structured, implemented, and scaled. **Methods and analysis:** The protocol was developed using the guidance for scoping reviews in the Joanna Briggs Institute manual and the PRISMA extension for scoping reviews checklist. A systematic search for published and unpublished literature will be conducted using the following databases: (1) MEDLINE; (2) PsycINFO; (3) HMIC; (4) EMBASE; and (5) ProQuest. To be included, documents must describe and/or evaluate an early intervention service for adolescents or adults with a non-psychotic mental health disorder. There will be no restrictions on publication type, study design, and date. Title and abstract, and full-text screening will be completed by one reviewer, with a proportion of articles screened in duplicate. Data analysis will primarily involve a qualitatively summary of the early intervention literature, the characteristics of early intervention services, and key findings relating to their evaluation and implementation. **Ethics and dissemination:** The synthesis of published and unpublished articles will not require ethical approval. The results of this scoping review will be published in a peer-reviewed journal and disseminated via social media, conference presentations, and other knowledge translation activities.

Keywords: early intervention, mental health disorders, service design, implementation

For peer review only

ARTICLE SUMMARY

Strengths and limitations of this study

- This scoping review will provide a comprehensive overview of both published and unpublished literature for the emerging research field of early intervention services for non-psychotic mental health disorders.
- The review will be conducted according to the standardised methodology outlined in the Joanna Briggs Institute manual and using the PRISMA checklist for scoping reviews.
- Part of the screening and charting process will be completed in duplicate to ensure reliability of these methods.
- Only articles written in English, German, French and Spanish will be included, the review may therefore be biased.

For peer review only

INTRODUCTION

Early intervention is widely perceived as beneficial in medicine and refers to the early detection and initiation of stage-specific treatment.[1] Pro-active treatments matched to the stage of illness can limit or even avert unfavourable outcomes, reducing the need for costly and more invasive treatments in the future.[2,3] Despite such promise, early intervention approaches have been slow to gain momentum in mental health.[4,5] Mental illnesses are a major contributor to mortality and disability worldwide, particularly for young people.[6-8] The typical age of onset for mental disorders is adolescence and early adulthood (12-30 years old), a period of marked social, psychological, and biological change.[9,10] A delay in or lack of access to effective treatments during this time could disrupt key developmental milestones and have long-lasting effects on health, social, and occupational trajectories.[11]

Service provision does not match the topography of onset or burden of disease associated with mental disorders, even in relatively well-developed health systems.[12] Globally, access to evidence-based care is poor, and even for those that do access it, this is often after lengthy delays.[13-15] The duration of untreated illness (DUI), defined as the period between the onset of psychiatric disorder and the initiation of treatment, ranges from 1-2 years for psychosis to 10 years for obsessive-compulsive disorder (OCD).[16-19] Over time, mental disorders can become more entrenched through functional deterioration, neuroadaptation, and habitual behaviour patterns.[20-23] Indeed, a longer DUI is associated with worse symptomatic and functional outcomes, and a lower treatment response across diagnostic groups.[19,24-27] More worryingly, young people, the group at highest risk for psychiatric difficulties, tend to have the worst access to timely care.[13,18,28-30]

Together, such findings provide a compelling case for establishing early intervention services that match the developmental needs and symptomatic profile of individuals with recent-onset mental disorders.[4,14] The greatest strides in early intervention have been made within psychosis. Over the past 30 years, early intervention for psychosis (EIP) has gained tremendous support from researchers and healthcare professionals worldwide.[14] EIP services have two fundamental aims: to reduce the duration of untreated psychosis, and to provide evidence-based, stage-specific treatment.[31] EIP services use a clinical staging approach to map the extent of illness progression from early pre-symptomatic risk to severe and enduring, enabling a prevention orientated framework that matches the intensity of treatment to the level of need.[32,33] A comprehensive body of high-quality research now shows that compared to standard care, multi-component EIP services are associated with a reduction in symptom severity, relapse rates and hospitalisation risk, as well as improved

For peer review only

global functioning and quality of life.[34] Moreover, consistent evidence suggests that EIP services are a cost-effective alternative to standard care.[35] There has been a recent surge in papers calling for early intervention approaches to be broadened to other diagnostic groups, including major depression,[36] OCD,[22] eating disorders,[37] and bipolar disorder.[38] Preliminary evidence from services for recent-onset eating and mood disorders demonstrate significant improvements in symptoms, reduced hospital (re)admissions, and most importantly, high levels of patient satisfaction.[39-42]

The utility of focusing exclusively on discrete diagnostic categories in the delivery of early intervention specifically, and mental health care more generally has, however, been questioned.[32,43] The early stages of mental disorder are often characterised by fluctuating patterns of specific and non-specific subthreshold symptoms, diagnostic instability, and comorbidity.[44,45] A single-disorder focus could result in these earlier presentations of illness being excluded.[46] A transdiagnostic approach, consistent with evidence for pluripotent models of clinical staging, has been put forward as a necessary solution to address this problem.[32,47,43,48] The recognition of the need to broaden the early intervention paradigm has led to the development of several integrated youth mental health hubs.[49,50] These hubs act as entry-level services for young people irrespective of diagnosis, and typically provide a comprehensive package of low-intensity mental, physical, and social care support in community settings. Young people tend to rate these services positively and between 52-68% of young people experience improvements in symptoms and functioning. However, a proportion of individuals with more severe symptoms do not seem to benefit from these services and rigorous outcome research for youth hubs is limited.[50,51]

Although the role of early intervention in reducing distress and functional impairment seems obvious, the evidence-base for these services is incomplete and much more work needs to be done.[14,22] There is limited prospective evidence evaluating the utility of these services for non-psychotic disorders, it is unclear to what extent the findings from psychosis would translate to other diagnostic groups. There is also a lack of research evaluating the feasibility or the implementation of services in clinical settings.[51] Moreover, even within psychosis, further research is needed to determine how long EIP services should be provided, whether it is the reduction in DUI or other components of EIP services that account for the improved outcomes, and whether outcomes would be similar with other service structures and models.[52,53] An ever-growing population accompanied by reducing health budgets, creates an environment where only services that demonstrate effectiveness, economic viability and sustainability receive funding.[54] It is therefore imperative to develop a

For peer review only

rigorous evidence-base to refine, adapt and evaluate early intervention services for non-psychotic disorders, with a particular focus on identifying the “active ingredients” of such services and the most effective methods for widespread scaling and implementation.

The primary objective of this review is to provide a baseline characterisation of the differing ways in which early intervention services are structured and implemented for non-psychotic mental health disorders. The emerging literature for non-psychotic disorders is heterogenous and dispersed, with distinct streams of research developing in disciplinary silos. The aim of this review is to draw together these streams to facilitate collaboration and cross-disciplinary learning and discourse. By synthesising the field and highlighting commonalities and differences, we hope that a broad set of common principles for early intervention services will emerge. This review, in conjunction with reviews in psychosis, will help set the stage for a more unified approach to expanding and refining early intervention services for psychiatric disorders. Here, we focus exclusively on disorders that tend to emerge in adolescence and adulthood rather than in childhood. Neurodevelopmental disorders typically use a very different approach to early intervention than adolescent- and adult-onset disorders (e.g. intervening in infancy).[55] A scoping review methodology was selected for this review as early intervention is an emerging, dispersed and heterogenous research area and is therefore not amenable to the narrower aims of a traditional systematic review.[56,57] Given that this is a relatively new research area, we sought to map all the available evidence within this field rather than only the best available evidence (e.g. randomised controlled trials).[58]

RESEARCH QUESTIONS

1. What is the extent, range, and nature of the literature on early intervention services for adolescents and adults with non-psychotic mental health disorders?
2. What are the characteristics of early intervention services and care pathways?
    - Are there any similarities and/or differences across early intervention services provided for each diagnosis and transdiagnostically?
3. Are there any factors that influence the implementation of early intervention services (i.e. barriers and facilitators to implementation)?
4. Do early intervention services reduce DUI, improve the course and outcome of mental disorders or minimise the disruption to psychosocial development and function?

METHODS AND ANALYSIS

For peer review only

The PRISMA extension for scoping reviews (PRISMA-ScR) checklist,[57] and the scoping review framework outlined in the Joanna Briggs Institute (JBI) Reviewer's Manual[59] were used to guide the development of this protocol. A copy of the PRISMA-ScR checklist can be seen in Table 1.

Table 1. PRISMA-ScR checklist

SECTION	ITEM	PRISMA-ScR CHECKLIST ITEM
**TITLE**		
Title	1	Identify the report as a scoping review.
**ABSTRACT**		
Structured summary	2	Provide a structured summary that includes (as applicable): background, objectives, eligibility criteria, sources of evidence, charting methods, results, and conclusions that relate to the review questions and objectives.
**INTRODUCTION**		
Rationale	3	Describe the rationale for the review in the context of what is already known. Explain why the review questions/objectives lend themselves to a scoping review approach.
Objectives	4	Provide an explicit statement of the questions and objectives being addressed with reference to their key elements (e.g., population or participants, concepts, and context) or other relevant key elements used to conceptualize the review questions and/or objectives.
**METHODS**		
Protocol and registration	5	Indicate whether a review protocol exists; state if and where it can be accessed (e.g., a Web address); and if available, provide registration information, including the registration number.
Eligibility criteria	6	Specify characteristics of the sources of evidence used as eligibility criteria (e.g., years considered, language, and publication status), and provide a rationale.

For peer review only

SECTION	ITEM	PRISMA-ScR CHECKLIST ITEM
Information sources*	7	Describe all information sources in the search (e.g., databases with dates of coverage and contact with authors to identify additional sources), as well as the date the most recent search was executed.
Search	8	Present the full electronic search strategy for at least 1 database, including any limits used, such that it could be repeated.
Selection of sources of evidence†	9	State the process for selecting sources of evidence (i.e., screening and eligibility) included in the scoping review.
Data charting process‡	10	Describe the methods of charting data from the included sources of evidence (e.g., calibrated forms or forms that have been tested by the team before their use, and whether data charting was done independently or in duplicate) and any processes for obtaining and confirming data from investigators.
Data items	11	List and define all variables for which data were sought and any assumptions and simplifications made.
Critical appraisal of individual sources of evidence§	12	If done, provide a rationale for conducting a critical appraisal of included sources of evidence; describe the methods used and how this information was used in any data synthesis (if appropriate).
Synthesis of results	13	Describe the methods of handling and summarizing the data that were charted.
**RESULTS**		
Selection of sources of evidence	14	Give numbers of sources of evidence screened, assessed for eligibility, and included in the review, with reasons for exclusions at each stage, ideally using a flow diagram.
Characteristics of sources of evidence	15	For each source of evidence, present characteristics for which data were charted and provide the citations.

For peer review only

SECTION	ITEM	PRISMA-ScR CHECKLIST ITEM
Critical appraisal within sources of evidence	16	If done, present data on critical appraisal of included sources of evidence (see item 12).
Results of individual sources of evidence	17	For each included source of evidence, present the relevant data that were charted that relate to the review questions and objectives.
Synthesis of results	18	Summarize and/or present the charting results as they relate to the review questions and objectives.
**DISCUSSION**		
Summary of evidence	19	Summarize the main results (including an overview of concepts, themes, and types of evidence available), link to the review questions and objectives, and consider the relevance to key groups.
Limitations	20	Discuss the limitations of the scoping review process.
Conclusions	21	Provide a general interpretation of the results with respect to the review questions and objectives, as well as potential implications and/or next steps.
**FUNDING**		
Funding	22	Describe sources of funding for the included sources of evidence, as well as sources of funding for the scoping review. Describe the role of the funders of the scoping review.

JBI = Joanna Briggs Institute; PRISMA-ScR = Preferred Reporting Items for Systematic reviews and Meta-Analyses extension for Scoping Reviews.

* Where *sources of evidence* (see second footnote) are compiled from, such as bibliographic databases, social media platforms, and Web sites.

† A more inclusive/heterogeneous term used to account for the different types of evidence or data sources (e.g., quantitative and/or qualitative research, expert opinion, and policy documents) that may be eligible in a scoping review as opposed to only studies. This is not to be confused with *information sources* (see first footnote).

‡ The frameworks by Arksey and O'Malley (6) and Levac and colleagues (7) and the JBI guidance (4, 5) refer to the process of data extraction in a scoping review as data charting.

§ The process of systematically examining research evidence to assess its validity, results, and relevance before using it to inform a decision. This term is used for items 12 and 19 instead of "risk of bias" (which is more applicable to systematic reviews of interventions) to include and acknowledge the various sources of evidence that may be used in a scoping review (e.g., quantitative and/or qualitative research, expert opinion, and policy document).

For peer review only

Eligibility criteria

Documents will be included if they: (1) Describe and/or evaluate an early intervention service for non-psychotic mental health disorders (*concept*) based in any type of healthcare facility (i.e. hospitals, day services, and community settings) and in any geographic area (*context*). Here, early intervention refers to a structured programme of care delivered by a stand-alone team or teams integrated into mental health services that provide treatment for individuals with recent-onset subthreshold or threshold disorders. The level of care can vary from low-intensity techniques of signposting, psychoeducation, and self-help resources all the way through to specialised multi-disciplinary teams and complex high intensity interventions. (2) Describe and/or evaluate an early intervention service for adolescents (≥ 10-17 years) or adults (> 18 years) with a recent-onset subthreshold or threshold mood disorder, anxiety disorder, eating disorder, personality disorder, impulse control or substance use disorder, and/or somatoform disorder (*types of participants*). Transdiagnostic early intervention services and early intervention services for comorbid/concurrent disorders will be included provided that at least one of the diagnoses is listed in the previous sentence. (3) Mixed child and adolescent services will be included, where feasible, only information relevant for the adolescent portion of the services will be charted. (4) All document types and study designs are eligible for inclusion: randomised controlled trials, non-randomised studies, observational studies, qualitative studies, reviews, ongoing trials, protocols, theoretical papers, grey literature, editorials, opinions pieces, and expert consensus statements (*types of studies*).

Documents will be excluded if they: (1) Describe a primary prevention programme based in educational establishments, high-risk groups (e.g. athletes), or in the general population, (2) Describe a parent only intervention, (3) Describe a specific intervention (e.g. type of CBT) that is not attached to a service, (4) Primarily or only focus on early intervention for a physiological or medical condition, schizophrenia spectrum and other psychotic disorders, and/or neurodevelopmental disorders.

Search strategy

A comprehensive literature search will be conducted from inception on PsycINFO, MEDLINE, EMBASE, and HMIC. ProQuest databases will also be searched for grey literature (i.e. conference papers and proceedings, theses, government publications). The search is completed in three stages. First, an initial limited search was conducted in MEDLINE using the terms "early intervention" and "mood disorder" or "anxiety disorder" or "eating disorder" or "personality disorder" or "impulse control disorder" or "substance use disorder" or

For peer review only

"somatoform disorder". The initial limited search was conducted by KR in April 2019 to identify keywords and subject headings to generate a search strategy. Different combinations of keywords and subject headings were trialled in MEDLINE, and key papers from the early intervention field were used as indicators for the sensitivity of the search strategy. The preliminary search strategy was developed by KR and reviewed by AA, KA, and US. An iterative process was used to balance the sensitivity and specificity. The MEDLINE-specific search strategy returns 3,545 documents before de-duplication and is outlined in Table 2.

In the second stage, all databases will be searched using the MEDLINE search strategy. The search strategy will be tailored to each database. The search for scoping reviews are more iterative than systematic reviews, it is therefore feasible that as the reviewers become more familiar with the literature that additional search terms and sources may be identified. The final stage involves identifying additional articles by searching the reference lists of included articles. Studies not reported in English, German, French, and Spanish will be excluded from the review during the screening and eligibility assessment. No date limits will be applied to the search. References will be imported to the EndNote x8 reference manager.

Table 2. MEDLINE search strategy

	**Query**	**Results**
#1	exp Early Medical Intervention [MeSH term]/ or (early intervention* or early-intervention*).tw	19623
#2	exp Mood Disorders [MeSH term]/ or Bipolar Disorders [MeSH term]/ or (mood disorder* or affective disorder* or depressi* or dysthymi* or bipolar*).tw	453041
#3	#1 AND #2	1616
#4	exp Anxiety Disorders [MeSH term]/ or (anxiety disorder* or neurotic disorder* or agoraphobi* or obsessive-compulsive disorder* or OCD or panic disorder* or phobic disorder* or post-traumatic stress disorder* or post traumatic stress disorder* or PTSD or generalised anxiety disorder* or social phobia).tw	119604
#5	#1 AND #4	560
#6	exp "Feeding and Eating Disorders" [MeSH term]/ or (eating disorder* or anorexi* or bulimi* or binge-eating* or binge eating* or (eating disorder not otherwise specified) or EDNOS or (other specified feeding or eating disorder) or OSFED).tw	56480
#7	#1 AND #6	199

For peer review only

#8	exp Substance-Related Disorders [MeSH term]/ or exp "Disruptive, Impulse Control, and Conduct Disorders" [MeSH term]/ or (((substance-related or alcohol or opioid or morphine or marijuana or heroin or cocaine or amphetamine or cannabis) adj1 (disorder* or illness* or dependence or abuse or misuse)) or (impulse control disorder*) or conduct disorder* or fire setting behaviour* or gambling or trichotillomania).tw	295108
#9	#1 AND #8	924
#10	exp Somatoform Disorders [MeSH term]/ or (somatoform or somatoform disorder* or somati#ation or body dysmorphi* or conversion disorder* or hypochondri*).tw	25487
#11	#1 AND #10	38
#12	exp Personality Disorders [MeSH terms]/ or (personality disorder* or antisocial personality disorder* or anti-social personality disorder* or borderline personality disorder* or emotionally unstable personality disorder* or obsessive-compulsive personality disorder* or dependent personality disorder* or histrionic personality disorder* or narcissistic personality disorder* or avoidant personality disorder* or paranoid personality disorder* or schizoid personality disorder* OR schizotypal personality disorder*).tw	47019
#13	#1 AND #12	208

For peer review only

Study selection process

The title and abstract screening in the second stage of the search will be completed by one reviewer with a portion of the articles being screened in duplicate to ensure reliability (25%). Retrieved full-texts will also be screened by one reviewer with a sample of full-text documents (25%) being screened in duplicate for reliability. The eligibility criteria will be applied to each document on a case-by-case basis to determine eligibility for inclusion. Discrepancies between reviewers will be resolved by discussion and if necessary other members of the review team will be consulted.

Data items and charting

A standardised data charting form developed by the study team will be used to chart the data from eligible studies (see Table 3 for a description of each data item). The data charting form was developed using the template from the JBI manual and by drawing on recent reviews of youth service models.[50, 51] Each section of the data charting form was developed to address one of the four research questions. The 'Document Details' section which provides descriptive information on document type, author(s), publication date, title and aim/purpose of document will be used to evaluate the extent, nature, and range of the literature on early intervention services (question 1). The second section 'Characteristics of Early Intervention Service' will address the second question as key characteristics of the services, namely the population, setting, structure, and interventions used in early intervention services will be charted (question 2). The 'Outcome Research' section will be used to answer questions 3 and 4 as any data related to implementation, effectiveness, or efficacy will be charted (question 3 & 4). Similar to the full-text screening, one reviewer will chart the majority of the documents with only a portion (25%) of the documents being charted in duplicate to ensure reliability. A small selection of documents will be charted by both reviewers at the outset to ensure that there is clarity and consistency in the use of the data charting form. Where there is more than one paper on the same service model, information will be pooled across the papers to provide the most detailed description of the model and any available evidence.

Table 3. Draft data charting form

**Data Item**	**Description of Item**
**Document Details**	
Type of document	The type of document can include but will not be limited to published or unpublish primary research, any type of review, protocols, theoretical paper, guidelines, opinion pieces, editorials, and expert consensus papers.
Author(s)	List of authors
Year of publication	Year of publication
Title	Title of document
Journal	The title of the scientific journal (for published documents only)
Country of origin	Country where the document originates

For peer review only

Aim/purpose of document	Summary of the aim/purpose of the document
Study design	For published or unpublished research papers, the design of the study as reported in the paper. Includes but is not limited to randomised controlled trials, pre-post design, historical controlled trial, prospective or retrospective cohort studies, cross-sectional, and case series/study.
Study methodology	The methodological framework: qualitative, quantitative, or mixed methods.
**Characteristics of Early Intervention Service**	
Name of service	The name of the early intervention service/program.
Year established	The year the early intervention service was established.
Location	The country and region in which the early intervention service was implemented.
Population	The population for which the service was designed for. This item will include details such as age, diagnosis, duration of illness, and illness severity.
Setting	The physical setting in which the early intervention service is based. This includes but is not limited to community centres, primary care, outpatient clinics, and inpatient wards. Early intervention services can occupy more than one of these settings.

For peer review only

Service providers	A description of who provides the service and their role, includes but is not limited to social workers, youth workers, peer support workers, nurses, clinical or counselling psychologists, and psychiatrists.
Service structure/process	A description of the service structure and administrative processes includes but is not limited to 'service within a service' models, stand-alone multi-disciplinary team models, 'hub' and 'spoke' models, and process variables such as specific wait time targets.
Access to service	Methods for accessing the early intervention service, includes but is not limited to active engagement and outreach through schools, colleges and youth clubs, referral from primary care, self-referral, and drop-in.
Services and interventions	A description of the types of services and interventions provided, includes but is not limited to psychoeducation, online self-help and self-management support, psychological therapies (e.g. CBT, brief therapy), sexual health and family planning, health promotion, social services, peer support, and crisis intervention and management.
Clinical staging	Whether a clinical staging approach was used to inform the design, evaluation, or implementation of the service.
**Outcome Research**	
Participants	Details related to the participants included in the study. This will include information related to sample size, diagnosis, age, sex, and inclusion/exclusion criteria.

For peer review only

Comparator data or standard care	Description of comparator data or the care provided to a control group.
Outcomes and time-points	Description of the qualitative and quantitative outcomes and the time points of data collection. This will include standardised clinical assessments, and self-report measures as well as implementation outcomes, such as measures of acceptability, feasibility, adoption, fidelity, and sustainment.
Key results/findings	An outline of the key results and findings reported in the document. This includes quantitative outcomes such as changes in symptoms, engagement, and patient satisfaction, as well as qualitative outcomes, such as, descriptions of barriers and facilitators to implementation.

For peer review only

Critical appraisal

The lack of critical appraisal tools in scoping reviews has been highlighted as one of the primary limitations of this knowledge synthesis method.[60] Critical appraisal can facilitate the interpretation of reviews by identifying the relative strengths and weaknesses of the included articles and identifying gaps in the research field. However, formal evaluations of methodological quality for scoping reviews can be challenging given the diversity of study designs and the volume of included literature.[61] Given the range of study designs, a two-stage assessment of methodological quality will be conducted for this review. First, each study will be ranked using the Joanna Briggs Institutes Levels of Evidence for Effectiveness from high (Level 1) to low (Level 5) (Level 1 – Experimental Designs; Level 2 – Quasi-experimental Designs; Level 3 – Observational - Analytical; Level 4 – Observational - Descriptive; Level 5 – Expert Opinion and Bench Research).[62] Once stratified according to the level of evidence, the quality of the studies within each stratum will be evaluated using the Joanna Briggs Institute Critical Appraisal tools.[63] Additionally, the generalisability and real-world applicability (external validity) of the included studies will be evaluated against the domains of the RE-AIM

(Reach, Effectiveness, Adoption, Implementation, and Maintenance) framework. A modified version of a RE-AIM framework rating system developed by Gaglio and colleagues will be used in the current study.[64] The modified rating system can be seen in Table 4. Each document will be given a rating ranging from 1 (limited generalisability or no information) to 3 (generalisable/pragmatic or information to enable generalisation) on six key domains: Participant Representativeness, Setting Representativeness, Outcome Representativeness, Fidelity/Adaptation, Cost/Feasibility of Intervention, and Sustainment. A narrative summary of the methodological quality will be provided alongside quantitative values for each domain of the RE-AIM framework. A portion of the included articles will be appraised in duplicate.

Synthesis of results

The search results will be reported using a flow diagram to clearly detail the review decision process, indicating the number of citations screened, duplicates removed, study selection, and full texts retrieved. The characteristics of the included studies will be presented in an informative table with a narrative and quantitative (e.g. frequencies) summary in text. Figures will be used to display the distribution of documents over time and across diagnoses. Descriptions of the early intervention services will be reported for each diagnostic group and transdiagnsotically along with any evidence supporting the services and barriers and facilitators to implementation. An aggregated summary of early intervention services with descriptions of common themes and differences across the services will be provided. An effort will be made to identify gaps in knowledge to inform the direction of future research.

Patient and public involvement

No patients or public were involved in the development of this protocol.

ETHICS AND DISSEMINATION

This review contributes to the growing body of research for early intervention initiatives in mental health by mapping the existing literature on early intervention services for non-psychotic mental health disorders. Through the publication of the results and dissemination via social media and conference presentations, the results will hopefully provide a timely foundation for cross-disciplinary discourse and early intervention service development and research. The results of this review may inform the design of new services and policies to support them. The synthesis of existing knowledge will not require ethical approval.

For peer review only

Table 4. Summary of Reach, Effectiveness, Adoption, Implementation, and Maintenance (RE-AIM) framework criteria

Reach (Participant Representativeness)	The representativeness of individuals enrolled in the study to the characteristics of the intended population.<br>1 = Limited generalisability: highly selected subsample that is not typical of the intended population, high number of exclusionary criteria, and/or a recruitment strategy that is likely to result in a biased sample.<br>2 = Moderately generalisable: participants match intended population on key characteristics (e.g. sex/gender, diagnosis, age), but are still a selected subsample due to exclusion criteria and recruitment strategies.<br>3 = Generalisable: participants are typical of the intended population, limited or no exclusion criteria, and/or recruitment strategies is not selective and are unlikely to result in a biased sample.
Effectiveness (Outcome Representativeness)	Measured outcomes are important and meaningful to all stakeholders involved, including potential negative effects, quality of life, and economic outcomes.<br>1 = Limited generalisability: primary outcomes restricted to an estimate of the overall effect of the intervention on a single metric of health, limited attention to process outcomes, quality of life, patient and staff satisfaction, patient engagement, unintended harms, or functional rehabilitation.<br>2 = Moderate generalisability: primary outcomes focus on overall effect of intervention on health, some inclusion of measures that are meaningful to stakeholders or process outcomes.<br>3 = Generalisable outcomes: primary outcomes include mix of impact of intervention on health and outcomes that are meaningful to patients and other stakeholders (including qualitative evaluations), explicit discussion around prevention of harms to participants, process outcomes, patient engagement, acceptability and satisfaction.
Adoption (Setting Representativeness)	The representativeness of settings and the individuals within those settings who deliver the program.

	1 = Limited generalisability: highly selected settings and staff and/or only includes 'best' sites and staff, i.e. well-resourced, credentialed or seasoned interventionists, many exclusion criteria; or limited information to determine context of study or intervention.<br>2 = Moderate generalisability: intervention tested in contexts outside of 'best' sites and staff, but adoption is still limited to selected settings that are well-resourced with some expertise in intervention trials.<br>3 = Generalisable: sites and staff are randomly selected, few or no exclusion criteria, and/or trialled in diverse settings.
Implementation (Fidelity/Adaptation, & Cost/Feasibility)	Fidelity to the intervention and adaptations made to intervention during study/program.<br>1 = Limited information on the implementation: no details on adaptation to local context, no details related to core element of interventions, or an evaluation of the consistency of implementation across settings, staff, and patients<br>2 = Moderate reporting of fidelity/adaptations: core elements described but details missing, or fidelity was monitored but no details on measurement tools.<br>3 = Detailed report of modifications made, adaptations to local context, and rationale for modification, an outline of core elements and evaluation of the fidelity to core elements of the model.
	The cost of the intervention in terms of time and money.<br>1 = No details on time, cost, and resources, no efforts to contain costs, and use of state-of-the-art resources and procedures such that costs of intervention are likely to be high.<br>2 = Details on time, cost, and resources is still limited but more than for a rating of 1. The intervention has minimal impact on time, cost, and resources.<br>3 = Explicit efforts to contain costs and to make the intervention feasible in low resource settings.

For peer review only

Maintenance (Sustainment)	The extent to which an intervention becomes institutionalized or part of the routine organizational practices and policies and the extent to which behaviour is sustained for more than 6 months.<br>1 = Limited sustainability efforts or details of such efforts: no report of efforts to continue an intervention after the completion of study, or no reports of continued use.<br>2 = Moderate sustainment: limited discussion regarding the sustainability of an intervention, some evidence of continued use.<br>3 = Sustainment: long-term outcomes reported, explicit plans for handing off intervention to setting/sites, details of methods to encourage sustainable implementation or embedding within routine organisational practices and policies, or evidence of sustained use for 6 months or more.

For peer review only

Author contributions All authors contributed to the development of this protocol. KR drafted the manuscript and search strategy. AA, KA, and US reviewed the search strategy and the draft manuscripts. KR incorporated the feedback from the authors. All authors read and approved the final manuscript.

Funding statement KR and AA are supported by the Health Foundation Scaling Up programme. US is supported by a National Institute of Health Research (NIHR) Senior Investigator Award and receives salary support from the NIHR Mental Health Biomedical Research Centre at the South London and Maudsley NHS Foundation Trust and King's College London. KA is supported by the NHS Innovation Accelerator programme. The views expressed are those of the author(s) and not necessarily those of the NHS, the NIHR or the Department of Health.

Competing interest statement None declared.

Patient consent Not required.

For peer review only

REFERENCES

1. McGorry PD. Is early intervention in the major psychiatric disorders justified? Yes. *BMJ* 2008;337:a695. doi: 10.1136/bmj.a695
2. Gillies CL, Abrams KR, Lambert PC, et al. Pharmacological and lifestyle interventions to prevent or delay type 2 diabetes in people with impaired glucose tolerance: systematic review and meta-analysis. *BMJ* 2007;334(7588):299. doi: 10.1136/bmj.39063.689375.55
3. Shah C, Arthur DW, Wazer D, et al. The impact of early detection and intervention of breast cancer-related lymphedema: a systematic review. *Cancer Med J* 2016;5(6):1154-62. doi: 10.1002/cam4.691
4. McGorry PD, Mei C. Early intervention in youth mental health: progress and future directions. *Evid Based Ment Health* 2018;21(4):182. doi: 10.1136/ebmental-2018-300060
5. McGorry PD, Ratheesh A, O'Donoghue B. Early Intervention—An Implementation Challenge for 21st Century Mental Health CareEarly Intervention—An Implementation Challenge for 21st Century Mental Health CareEditorial. *JAMA Psychiatry* 2018;75(6):545-46. doi: 10.1001/jamapsychiatry.2018.0621
6. Patel V, Flisher AJ, Hetrick S, et al. Mental health of young people: a global public-health challenge. *Lancet (London, England)* 2007;369(9569):1302-13. doi: 10.1016/S0140-6736(07)60368-7
7. Rehm J, Shield KD. Global Burden of Disease and the Impact of Mental and Addictive Disorders. *Curr Psychiatry Rep* 2019;21(2):10. doi: 10.1007/s11920-019-0997-0
8. Vigo D, Thornicroft G, Atun R. Estimating the true global burden of mental illness. *Lancet Psychiatry* 2016;3(2):171-78. doi: 10.1016/S2215-0366(15)00505-2
9. de Girolamo G, McGorry PD, Sartorius N, editors. *Age of Onset of Mental Disorders*. Switzerland: Springer International, 2019.
10. Kessler RC, Angermeyer M, Anthony JC, et al. Lifetime prevalence and age-of-onset distributions of mental disorders in the World Health Organization's World Mental Health Survey Initiative. *World J Psychiatry* 2007;6(3):168-76.
11. Malla A, Shah J, Iyer S, et al. Youth Mental Health Should Be a Top Priority for Health Care in Canada. *Can J Psychiatry* 2018;63(4):216-22. doi: 10.1177/0706743718758968
12. Vigo DV, Kestel D, Pendakur K, et al. Disease burden and government spending on mental, neurological, and substance use disorders, and self-harm: cross-sectional,

For peer review only

ecological study of health system response in the Americas. *Lancet Public Health* 2019;4(2):e89-e96. doi: 10.1016/S2468-2667(18)30203-2

13. de Girolamo G, Dagani J, Purcell R, et al. Age of onset of mental disorders and use of mental health services: needs, opportunities and obstacles. *Epidemiol Psychiatric Sci* 2012;21(1):47-57. doi: 10.1017/S2045796011000746
14. McGorry PD. Early intervention in psychosis: obvious, effective, overdue. *J Nerv Ment Dis* 2015;203(5):310. doi: 10.1097/NMD.0000000000000284
15. Patel V, Saxena S, Lund C, et al. The Lancet Commission on global mental health and sustainable development. *Lancet (London, England)* 2018;392(10157):1553-98. doi: 10.1016/S0140-6736(18)31612-X
16. Albert U, Barbaro F, Bramante S, et al. Duration of untreated illness and response to SRI treatment in Obsessive-Compulsive Disorder. *Euro Psychiatry* 2019;58:19-26. doi: 10.1016/j.eurpsy.2019.01.017
17. Altamura AC, Buoli M, Albano A, et al. Age at onset and latency to treatment (duration of untreated illness) in patients with mood and anxiety disorders: a naturalistic study. *Int Clin Psychopharmacol* 2010;25(3):172-79.
18. Dagani J, Signorini G, Nielssen O, et al. Meta-analysis of the Interval between the Onset and Management of Bipolar Disorder. *Can J Psychiatry* 2017;62(4):247-58. doi: 10.1177/0706743716656607
19. Marshall M, Lewis S, Lockwood A, et al. Association Between Duration of Untreated Psychosis and Outcome in Cohorts of First-Episode Patients: A Systematic Review. *JAMA Psychiatry* 2005;62(9):975-83. doi: 10.1001/archpsyc.62.9.975
20. Anderson KK, Voineskos A, Mulsant BH, et al. The Role of Untreated Psychosis in Neurodegeneration: A Review of Hypothesized Mechanisms of Neurotoxicity in First-Episode Psychosis. *Can J Psychiatry* 2014;59(10):513-17. doi: 10.1177/070674371405901003
21. Currin L, Schmidt U. A critical analysis of the utility of an early intervention approach in the eating disorders. *J Ment Health* 2005;14(6):611-24. doi: 10.1080/09638230500347939
22. Fineberg NA, Dell'Osso B, Albert U, et al. Early intervention for obsessive compulsive disorder: An expert consensus statement. *Eur Neuropsychopharmacol* 2019;29(4):549-65. doi: 10.1016/j.euroneuro.2019.02.002

For peer review only

23. Schaffalitzky E, Leahy D, Armstrong C, et al. 'Nobody really gets it': a qualitative exploration of youth mental health in deprived urban areas. *Early Interv Psychiatry* 2015;9(5):406-11. doi: 10.1111/eip.12165

24. Altamura AC, Dell'Osso B, D'Urso N, et al. Duration of Untreated Illness as a Predictor of Treatment Response and Clinical Course in Generalized Anxiety Disorder. *CNS Spectr* 2008;13(5):415-22. doi: 10.1017/S1092852900016588 [published Online First: 11/07]

25. Altamura AC, Dell'Osso B, Berlin HA, et al. Duration of untreated illness and suicide in bipolar disorder: a naturalistic study. *Eur Arch Psychiatry Clin Neurosci* 2010;260(5):385-91.

26. Drancourt N, Etain B, Lajnef M, et al. Duration of untreated bipolar disorder: missed opportunities on the long road to optimal treatment. *Acta Psychiatr Scand* 2013;127(2):136-44. doi: 10.1111/j.1600-0447.2012.01917.x

27. Ghio L, Gotelli S, Cervetti A, et al. Duration of untreated depression influences clinical outcomes and disability. *J Affect Disord* 2015;175:224-28. doi: 10.1016/j.jad.2015.01.014

28. Cleary A, Nixon E, Fitzgerald M. Psychological health and well-being among young Irish adults. *Ir J Psychol Med* 2007;24(4):139-44. doi: 10.1017/S0790966700010569 [published Online First: 06/13]

29. Burgess PM, Pirkis JE, Slade TN, et al. Service use for mental health problems: findings from the 2007 National Survey of Mental Health and Wellbeing. *Aust N Z J Psychiatry* 2009;43(7):615-23. doi: 10.1080/00048670902970858

30. Weigel A, Rossi M, Wendt H, et al. Duration of untreated illness and predictors of late treatment initiation in anorexia nervosa. *J Public Health* 2014;22(6):519-27. doi: 10.1007/s10389-014-0642-7

31. McGorry PD, Edwards J, Mihalopoulos C, et al. EPPIC: An Evolving System of Early Detection and Optimal Management. *Schizophr Bull* 1996;22(2):305-26. doi: 10.1093/schbul/22.2.305

32. Cross SP, Hickie I. Transdiagnostic stepped care in mental health. *Public Health Res Pract* 2017;27(2):1. doi: 10.17061/phrp2721712

33. McGorry PD, Hickie IB, Yung AR, et al. Clinical staging of psychiatric disorders: a heuristic framework for choosing earlier, safer and more effective interventions. *Aust N Z J Psychiatry* 2006;40(8):616-22. doi: 10.1111/j.1440-1614.2006.01860.x

For peer review only

34. Correll CU, Galling B, Pawar A, et al. Comparison of Early Intervention Services vs Treatment as Usual for Early-Phase Psychosis: A Systematic Review, Meta-analysis, and Meta-regression. *JAMA Psychiatry* 2018;75(6):555-65. doi: 10.1001/jamapsychiatry.2018.0623

35. Aceituno D, Vera N, Prina AM, et al. Cost-effectiveness of early intervention in psychosis: systematic review. *The British journal of psychiatry : the journal of mental science* 2019;215(1):388-94. doi: 10.1192/bjp.2018.298 [published Online First: 2019/01/31]

36. Davey CG, McGorry PD. Early intervention for depression in young people: a blind spot in mental health care. *Lancet Psychiatry* 2019;6(3):267-72. doi: 10.1016/S2215-0366(18)30292-X

37. Schmidt U, Brown A, McClelland J, et al. Will a comprehensive, person-centered, team-based early intervention approach to first episode illness improve outcomes in eating disorders? *The International journal of eating disorders* 2016;49(4):374-77. doi: 10.1002/eat.22519

38. Vieta E, Salagre E, Grande I, et al. Early Intervention in Bipolar Disorder. *Am J Psychiatry* 2018;175(5):411-26. doi: 10.1176/appi.ajp.2017.17090972

39. Brown A, McClelland J, Boysen E, et al. The FREED Project (first episode and rapid early intervention in eating disorders): service model, feasibility and acceptability. *Early Interv Psychiatry* 2018;12(2):250-57. doi: https://dx.doi.org/10.1111/eip.12382

40. Kessing LV, Hansen HV, Hvenegaard A, et al. Treatment in a specialised out-patient mood disorder clinic v. standard out-patient treatment in the early course of bipolar disorder: randomised clinical trial. *The British journal of psychiatry : the journal of mental science* 2018;202(3):212-19. doi: 10.1192/bjp.bp.112.113548 [published Online First: 01/02]

41. Osuch E, Vingilis E, Summerhurst C, et al. Process Evaluation of a Treatment Program for Mood and Anxiety Disorders Among Emerging Adults: Preentry Factors, Engagement, and Outcomes. *Psychiatr Serv* 2019;70(3):211-18. doi: 10.1176/appi.ps.201800219

42. McClelland J, Hodsoll J, Brown A, et al. A pilot evaluation of a novel First Episode and Rapid Early Intervention service for Eating Disorders (FREED). *European Eating Disorders Review* 2018;26(2):129-40.

For peer review only

43. McGorry PD, Ratheesh A, O'Donoghue B. Early Intervention-An Implementation Challenge for 21st Century Mental Health Care. *JAMA Psychiatry* 2018;75(6):545-46. doi: 10.1001/jamapsychiatry.2018.0621 [published Online First: 2018/05/26]

44. Iorfino F, Scott EM, Carpenter JS, et al. Clinical stage transitions in persons aged 12 to 25 years presenting to early intervention mental health services with anxiety, mood, and psychotic disorders. *JAMA Psychiatry* 2019 doi: 10.1001/jamapsychiatry.2019.2360

45. McGorry PD, Hartmann JA, Spooner R, et al. Beyond the "at risk mental state" concept: transitioning to transdiagnostic psychiatry. *World Psychiatry* 2018;17(2):133-42. doi: 10.1002/wps.20514 [published Online First: 2018/06/02]

46. Cross SP, Hermens DF, Scott EM, et al. A clinical staging model for early intervention youth mental health services. *Psychiatr Serv* 2014;65(7):939-43. doi: 10.1176/appi.ps.201300221 [published Online First: 2014/05/16]

47. Scott J, Leboyer M, Hickie I, et al. Clinical staging in psychiatry: a cross-cutting model of diagnosis with heuristic and practical value. *The British journal of psychiatry : the journal of mental science* 2018;202(4):243-45. doi: 10.1192/bjp.bp.112.110858 [published Online First: 01/02]

48. McGorry P, Nelson B. Why We Need a Transdiagnostic Staging Approach to Emerging Psychopathology, Early Diagnosis, and Treatment. *JAMA Psychiatry* 2016;73(3):191-2. doi: 10.1001/jamapsychiatry.2015.2868 [published Online First: 2016/01/15]

49. Lee VW, Murphy BP. Broadening the early intervention paradigm: a one stop shop for youth. *Early Interv Psychiatry* 2013;7(4):437-41. doi: 10.1111/eip.12055 [published Online First: 2013/05/21]

50. Hetrick SE, Bailey AP, Smith KE, et al. Integrated (one-stop shop) youth health care: best available evidence and future directions. *The Medical journal of Australia* 2017;207(10):S5-s18. doi: 10.5694/mja17.00694 [published Online First: 2017/11/14]

51. Settipani CA, Hawke LD, Cleverley K, et al. Key attributes of integrated community-based youth service hubs for mental health: a scoping review. *Int J Ment Health Syst* 2019;13(1):52. doi: 10.1186/s13033-019-0306-7

52. Behan C, Masterson S, Clarke M. Systematic review of the evidence for service models delivering early intervention in psychosis outside the stand-alone centre. *Early Interv Psychiatry* 2017;11(1):3-13. doi: 10.1111/eip.12334

53. Fusar-Poli P, McGorry PD, Kane JM. Improving outcomes of first-episode psychosis: an overview. *World J Psychiatry* 2017;16(3):251-65. doi: 10.1002/wps.20446

For peer review only

54. Stuckler D, Reeves A, Loopstra R, et al. Austerity and health: the impact in the UK and Europe. *Eur J Public Health* 2017;27(4):18-21. doi: 10.1093/eurpub/ckx167
55. Cioni G, Inguaggiato E, Sgandurra G. Early intervention in neurodevelopmental disorders: underlying neural mechanisms. *Dev Med Child Neurol* 2016;58(4):61-66. doi: 10.1111/dmcn.13050
56. Peters MDJ, Godfrey CM, Khalil H, et al. Guidance for conducting systematic scoping reviews. *Int J Evid Based Healthc* 2015;13(3):141-46. doi: 10.1097/XEB.0000000000000050
57. Tricco AC, Lillie E, Zarin W, et al. PRISMA Extension for Scoping Reviews (PRISMA-ScR): Checklist and ExplanationThe PRISMA-ScR Statement. *Ann Intern Med* 2018;169(7):467-73. doi: 10.7326/m18-0850
58. Murray A, Daines L, Archibald D, et al. The relationship and effects of golf on physical and mental health: a scoping review protocol. *Br J Sports Med* 2016;50(11):647. doi: 10.1136/bjsports-2015-095914
59. Peters MDJ, Godfrey C, McInerney P, et al. Chapter 11: Scoping Reviews. In: Aromataris E, Munn Z, eds. Joanna Briggs Institute Reviewer's Manual: The Joanna Briggs Institute, 2017.
60. Pham MT, Rajić A, Greig JD, et al. A scoping review of scoping reviews: advancing the approach and enhancing the consistency. *Res Synth Methods* 2014;5(4):371-85. doi: 10.1002/jrsm.1123
61. Levac D, Colquhoun H, O'Brien KK. Scoping studies: advancing the methodology. *Implement Sci* 2010;5(1):69. doi: 10.1186/1748-5908-5-69
62. Jordan Z, Lockwood C, Aromataris E, et al. The updated JBI model for evidence-based healthcare. 2016 [Available from: https://joannabriggs.org/jbi-approach.html#tabbed-nav=Levels-of-Evidence.
63. The Joanna Briggs Institute. Joanna Briggs Institute Critical Appraisal tools 2017 [Available from: https://joannabriggs.org/critical_appraisal_tools.
64. Gaglio B, Phillips SM, Heurtin-Roberts S, et al. How pragmatic is it? Lessons learned using PRECIS and RE-AIM for determining pragmatic characteristics of research. *Implement Sci* 2014;9(1):96. doi: 10.1186/s13012-014-0096-x

For peer review only
